# A meta-analysis of palliative treatment of pancreatic cancer with high intensity focused ultrasound

**DOI:** 10.1186/s40349-017-0080-4

**Published:** 2017-04-01

**Authors:** Susan Dababou, Cristina Marrocchio, Jarrett Rosenberg, Rachelle Bitton, Kim Butts Pauly, Alessandro Napoli, Joo Ha Hwang, Pejman Ghanouni

**Affiliations:** 1 Medical Student, Sapienza University of Rome, School of Medicine, V.le Regina Elena, 324, 00180 Rome, Italy; 2 Department of Radiology, Lucas Center for Imaging, Stanford University School of Medicine, 1201 Welch Road, Stanford, CA 94305 USA; 3 Department of Radiological Sciences, MRgFUS & Cardiovascular Imaging Unit, Sapienza University of Rome, School of Medicine, V.le Regina Elena, 324, 00180 Rome, Italy; 4 Gastroenterology Section, Harborview Medical Center, Bioengineering and Radiology, University of Washington, Box 359773, 325 Ninth Avenue, Seattle, WA 98104 USA

**Keywords:** Pancreatic cancer, High intensity focused ultrasound, HIFU, Pain palliation, Pain relief, Non-invasive treatment, Meta-analysis

## Abstract

**Background:**

Pancreatic adenocarcinoma is currently the fourth-leading cause of cancer-related death. Up to 60–90% of patients with advanced disease suffer cancer-related pain, severely impacting their quality of life. Current management involves primarily pharmacotherapy with opioid narcotics and celiac plexus neurolysis; unfortunately, both approaches offer transient relief and cause undesired side-effects. High intensity focused ultrasound (HIFU) is a non-invasive thermal ablation technique that has been used to treat pancreatic cancer. This meta-analysis aims to evaluate the role of HIFU in pain palliation of advanced unresectable pancreatic adenocarcinoma.

**Methods:**

An electronic search was performed in PubMed Medline database up to the end of July 2016, for unresectable pancreatic cancer pain palliation with HIFU. Pertinent studies were identified through the PubMed search engine using the following keywords: HIFU, pancreas, pancreatic cancer, pain and palliation. Additional studies were included after manual search of the selected bibliographies. Pain palliation results reported in each study were analyzed using a logit-transformed random-effects model using the inverse variance method, with the DerSimonian-Laird estimator for *τ* ^2^, and Cochran’s Q test for heterogeneity among studies. The I^2^ was calculated to assess the percentage of the total variability in the different effect size estimates that can be attributed to heterogeneity among the true effects. A rank correlation test of funnel plot asymmetry was done to assess possible publication bias.

**Results:**

The meta-analysis includes a total number of 23 studies with 865 patients, 729 with pancreatic cancer. The population enrolled ranges from 3 patients in the smallest series, up to 61 in the largest study. *τ* ^2^ (variance among studies) was 0.195, and I^2^ (percentage of variation among studies) was 40% (95% CI: 1–64%); the Q test p-value was 0.026, indicating significant heterogeneity among studies. Among 639 patients treated with HIFU, 567 complained of pancreatic pain before the treatment and 459 patients experienced partial or complete pain relief after treatment. The random effects estimate of the proportion of patients with pain reduction was 0.81 (95% CI: 0.76–86).

**Conclusions:**

HIFU appears to be an effective tool for pain palliation in advanced pancreatic cancer. Studies assessing treatment in patients with pancreatic adenocarcinoma are limited by factors such as small sample sizes and heterogeneity in clinical definitions and assessments. Prospective randomized and standardized studies are necessary to confirm the effectiveness of HIFU in relieving pain, and to evaluate for any potential impact on tumor control and patient survival.

## Background

Pancreatic cancer incidence is increasing worldwide with 53070 new cases and 41780 deaths estimated in 2016, and is currently the fourth cause of cancer-related death [1, 2]. It more frequently affects men between 65 and 84 years of age and occurs predominantly in the Western countries, where environmental factors may play an important role in the pathogenesis [3]. Ductal pancreatic adenocarcinoma, the most common histology, accounts for 85–90% of these cancers [4].

Despite advances in diagnostic methods and the development of new therapeutic approaches, the prognosis for pancreatic cancer has remained dismal over the past 40 years [4], with an overall 5-year survival rate of less than 8%, a median survival rate of 6–10 months for unresectable, locally advanced disease, and 3 to 6 months for patients with metastases [5]. The only possibility of cure is through surgery; however, due to the late appearance of symptoms, less than 20% of patients present with resectable disease at the time of diagnosis [6]. Moreover, mortality remains high even after surgery due to the high loco-regional recurrence rate and the tendency for early metastatic spread [7]. Considering the poor prognosis of these patients, the principal goals of pancreatic cancer therapy in advanced disease are to palliate symptoms and increase the overall survival. Throughout the illness and during end-of-life care, patients need comprehensive symptom control. Pain is common in patients with pancreatic cancer, and is reported by 60–90% of patients with advanced disease [8]. It is often described as dull pain, sometimes with colicky spasms, and is referred to the mid back or epigastric regions [9]. Gemcitabine-based chemotherapy and chemoradiation combinations produce a limited improvement in survival, but are not very effective in pain relief and are associated with high toxicity [10]. The current management of pancreatic cancer-related pain primarily involves pharmacotherapy with opioid narcotics and celiac plexus neurolysis. Unfortunately, opioid narcotics often cause undesired side effects ranging from mild constipation to altered mental status. In addition, some opioids can have a dysphoric effect that can significantly impact the patients’ quality of life [11]. Celiac plexus neurolysis is performed in patients who have severe intractable pain that is poorly controlled on opioids; however, the procedure is invasive, requiring endoscopic ultrasound or CT-guidance. Initial uncontrolled and retrospective case series suggested that partial or complete pain relief was achieved in 70–90% of patients undergoing neurolytic celiac plexus blockade (NCPB) [12]; however, a meta-analysis of five randomized controlled trials of NCPB demonstrated that the overall benefit was small, with only a 6% reduction in the mean visual analogue pain score compared to baseline [13]. Clearly, new methods are needed to both treat and palliate patients with advanced pancreatic cancer.

High intensity focused ultrasound (HIFU) is a non-invasive imaging-guided thermal ablation technique that uses an extracorporeal transducer to deliver ultrasound energy to induce an increase of temperature in a sharply demarcated region. Ultrasound or magnetic resonance imaging are used to assess the anatomy of the region for targeting and to provide real time feedback during ablation [14]. HIFU has a dual effect on the target tissue, inducing thermal and mechanical damage. During the treatment, the targeted tissue is heated to the 60–80 °C range within seconds, inducing liquefaction and coagulation necrosis in the targeted region, with the goal of thermal ablation of the tumor without affecting the surrounding healthy tissue [14, 15]. The temperature reached is not high enough to cause an immediate necrosis of the cells, but it induces first intracellular denaturation of protein, and thus of the stored pancreatic enzymes, followed by cellular degeneration and necrosis. This “thermal fixation” phenomenon potentially reduces the risk of pancreatitis as a complication of the procedure [15]. In addition to thermal effects, there are three mechanical effects associated with high intensity acoustic energy: cavitation, microstreaming and radiation force. Cavitation results from the oscillating motion of gas-filled bubbles (stable cavitation); these bubbles coalesce and collapse under higher ultrasound field energy, causing a shock wave confined to the microenvironment (inertial cavitation) [16, 17]. Microstreaming is the consequence of stable cavitation occurring close to fluids, producing shear stress that transiently damages the cell membrane [18]. Lastly, radiation force results from the absorption or reflection of the acoustic waves by the encountered medium and can result in cellular apoptosis [16, 17].

We aim to examine the current literature on the role of HIFU in pain palliation in advanced pancreatic cancer and to compare the methodologies used for treatment, with the goal of providing a comprehensive resource of comparable data for the design of future studies.

## Methods

### Article search

A systematic electronic search was performed using the PubMed Medline database through July 2016. The electronic system was interrogated with the following keywords: “HIFU” AND “Pancreatic cancer”, “HIFU” AND “Pain” AND “Pancreas”, “HIFU” AND “Pain” AND “Pancreatic Cancer”, “HIFU” AND “Palliation” AND “Pancreas”,” HIFU” AND “Palliation” AND “Pancreatic Cancer”. All variants of HIFU/high intensity focused ultrasound, pancreatic/pancreas, cancer/carcinoma, palliation/palliative were searched. Because some of the research published in this area was not written in English, it does not appear in PubMed; therefore, a manual search of the bibliographies of selected studies and reviews was completed to supplement the electronic search.

**Fig. 1:**
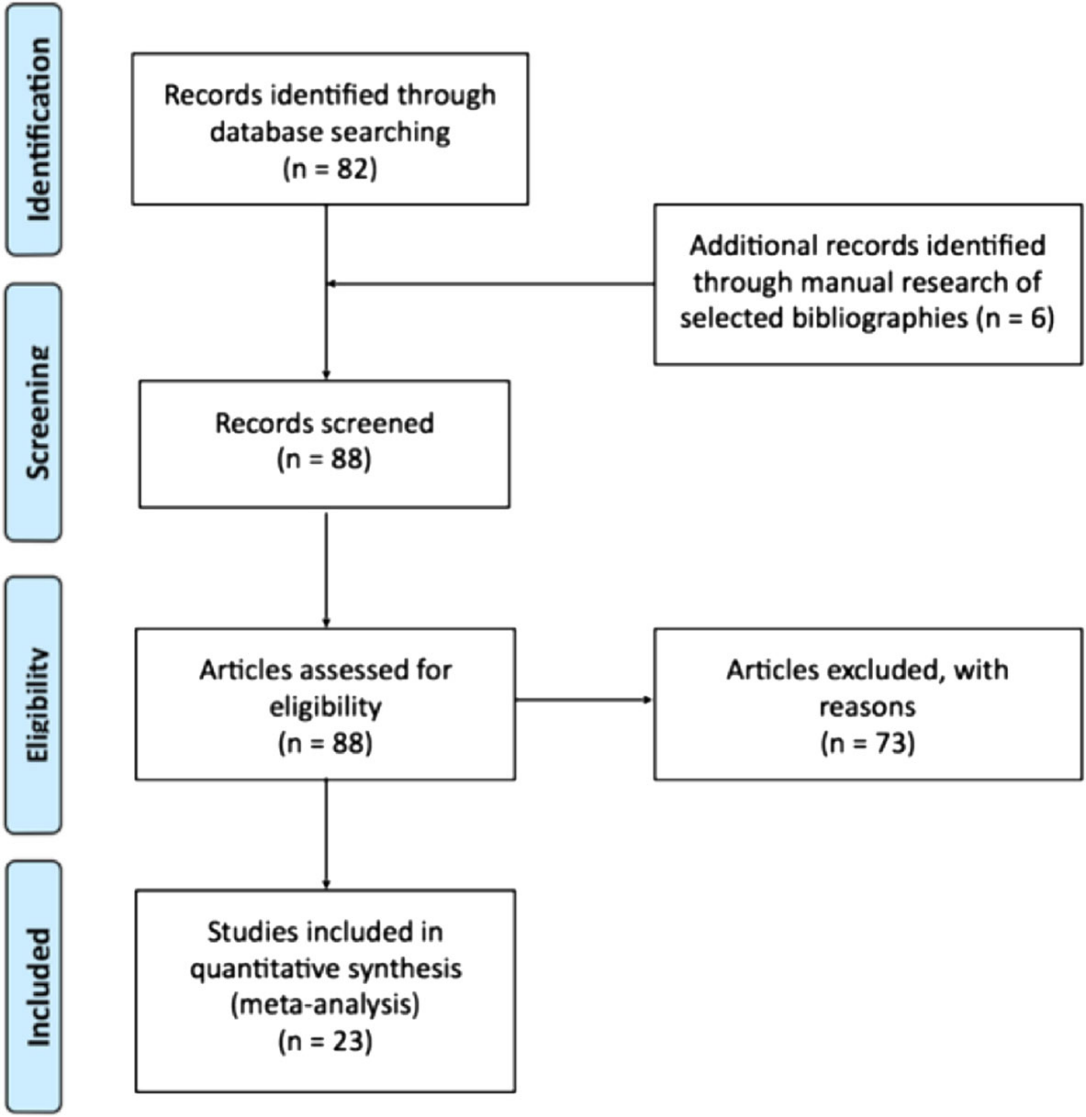
PRISMA Flowchart. PRISMA, Preferred Reporting Items for Systematic Reviews and Meta-analyses

The following exclusion criteria were applied: I) Reviews II) Studies not including pancreas III) Preclinical studies IV) Pain assessment not reported V) Reported imaging appearance or histology other than pancreatic adenocarcinoma VI) No primary pancreatic tumor VII) Studies with ≤ 2 patients VIII) Papers analyzing more than one type of malignancy in which data specific for pancreatic cancer related pain was not reported. Full-text articles were screened by SD, CM and PG. Translation of articles written in Chinese was provided by JHH. The flow of selection is described using the Preferred Reporting Items for Systematic Reviews and Meta-Analyses (PRISMA) model [19]. (Fig. 1)

### Statistical analysis

A meta-analysis of 23 studies on the therapeutic effect of HIFU on pancreatic cancer, defined as the proportion of patients having no or reduced pain post-procedure, was carried out. A logit-transformed random-effects model using the inverse variance method was used, with the DerSimonian-Laird estimator for *τ* ^2^, and Cochran’s Q test for heterogeneity among studies. The I^2^ was calculated to assess the percentage of the total variability in the different effect size estimates that can be attributed to heterogeneity among the true effects (substantial heterogeneity if I^2^ > 50%). A rank correlation test of funnel plot asymmetry was done to assess possible publication bias. All statistical analyses were done with R 3.1.2 and version 4.4.0 of the “meta” package (r-project.org).

## Results

### Search results and characteristics of the included studies

The primary electronic search identified 82 articles. Following the application of the exclusion criteria and the unavailability of one paper [20], 17 studies were selected. Six further studies were added from the manual research, five of which were translated from the Chinese language. A total of 23 studies were included in the statistical analysis. Studies were published between 2001 and 2016. Sixteen studies were from China, three from Italy, three from Germany, and one from Japan.

The demographic and clinical data are listed in Table 1. The studies include a total number of 865 patients; 729 had pancreatic cancer, of which 639 underwent HIFU treatment. The population enrolled ranges from 3 patients in the smallest series, up to 61 in the largest study. All 729 pancreatic cancers included were deemed unresectable. Three out of 20 papers were not limited to pancreatic cancer but also included other abdominal or pelvic malignancies. In 4 papers HIFU was combined with chemotherapy (2 with Gemcitabine, 1 with S-1 and in 1 is not specified), in 1 with radiotherapy and in the other 16 papers focused ultrasound was variably associated with prior chemotherapy and/or radiotherapy. In two clinical studies the patients were divided into two groups, comparing chemotherapy alone to a combination treatment including HIFU. MRI guidance was performed only in one study; the others used B-mode ultrasound.

**Table 1:** Characteristics of the included studies on HIFU therapy in pancreatic cancer

Author, Date	Type of Study	Number of total patients	Age (mean,*median)	Tumor Characteristics (#pt)			Image guidance	Treatment	HIFU Device	Other associated treatments (#pt)
				Stage	Location	Size				
Xiong, 2001 [53]	N/A	21	54	Stage III (12) Stage IV (9)	Head (8) Tail (13)	10x8x6 cm - 4x3x2 cm	US	HIFU	Pulsed wave HIFU, FEB-BY01 HIFU System	Failed surgery (5) Bile duct enterostomy (5)
Xu, 2003 [54]	N/A	37	62	Stage III (28) Stage IV (9)	Head (21) Body/Tail (11) Tail (5)	7x8x8 cm - 2x2x2 cm	US	HIFU	Pulsed wave HIFU, FEB-BY01 HIFU System	Enterocholecystotomy (21)
Yuan, 2003 [55]	N/A	40	64	N/A	Head (29) Body (8) Tail (3)	Average 5.4 cm, range 3.0–7.8 cm	US	HIFU	Pulsed wave HIFU, FEB-BY01 HIFU System	Obstructive jaundice surgically addressed prior to HIFU (20)
Gu, 2004 [21]	Retrospective	45 (38 treated)	55.5	Stage II (5) Stage III (30) Stage IV (10)	Head (20) Body (11) Tail (6) Head and Body (4) Tail and Body (3)	Tumor volume average 30–360 mm3	US	HIFU	Pulsed wave HIFU, FEB-BY01 HIFU System	Exploratory surgery (24) Bile duct enterostomy (22)
Li, 2004 [22]	N/A	102 (10 pancreatic cancers)	61	N/A	N/A	N/A	US	HIFU+ Chemotherapy	Pulsed wave HIFU, FEB-BY01 HIFU System	Chemotherapy
Wu, 2005 [32]	Prospective	8	62	Stage III (3) Stage IV (5)	Body (2) Head and Body (2) Tail and Body (4)	Diameter of primary tumor mean 5.89x5.40 cm	US	HIFU	Continuous wave HIFU, Model-JC HIFU System	Exploratory surgery (2) Chemotherapy (2) Local radiation therapy (1) IV Somatostatin (8)
Xie, 2008 [56]	N/A	16	50	N/A	N/A	N/A	US	HIFU	Continuous wave HIFU, HIFUNIT-9000 HIFU System	Chemotherapy (7)
Xiong, 2009 [44]	Retrospective	89	65	Stage II (4) Stage III (39) Stage IV (46)	Head (34) Tail and/or Body (55)	N/A	US	HIFU	Pulsed wave HIFU, FEB-BY01 HIFU System	Chemotherapy and/or radiotherapy before HIFU (39) Chemotherapy concurrent with HIFU (5) Biliary stent or surgery (26)
Zhao, 2010 [45]	Retrospective	39 (37 assessed)	55	Stage IIa (3) Stage IIb (5) Stage III (31)	Body (12) Head (27)	Longest diameter median 3.4 cm (range 1.7–8.5 cm)	US	HIFU+ Chemotherapy	Continuous wave HIFU, HIFUNIT-9000 HIFU System	Endoscopic biliary drainage with plastic stent (9) Percutaneous biliary drainage (4)
Orsi, 2010 [57]	Prospective	31 (6 pancreatic cancers)	64	N/A	N/A	N/A	US	HIFU	Continuous wave HIFU, Model-JC HIFU System	Chemotherapy and radiotherapy (6)
Wang, 2011 [33]	Prospective	40	57*	Stage III (13) Stage IV (27)	Head (9) Tail and/or Body (31)	Tumor size range 2–10 cm, median tumor size 4.3 cm	US	HIFU	Continuous wave HIFU, Model-JC HIFU System	Chemotherapy (28) Chemotherapy + radiotherapy (7) IV Somatostatin (40) Biliary bypass procedure (2) Endoscopic stenting (2) Cholecystojejunostomy (2)
Sung, 2011 [35]	Prospective	46	61	Stage III (18) Stage IV (28)	Head (17) Tail and/or Body (25) Tail (4)	Mean 4.2 +/- 1.4 cm, range 1.6–9.3 cm	US	HIFU	Continuous wave HIFU, Model-JC HIFU System	Pre HIFU Chemotherapy (20) (1 pt Surgical Resection) Chemoradiation therapy (10) Radiation therapy (3) After HIFU Chemotherapy (29) Chemoradiation (1) Duodenal stent (1)
Orgera, 2011 [58]	Prospective	22 (3 pancreatic cancers)	61	N/A	N/A	N/A	US	HIFU	Continuous wave HIFU, Model-JC HIFU System	Chemotherapy + radiotherapy (6) Alcohol ablation before HIFU (1)
Li, 2012 [59]	Retrospective	25	60	Stage III (12) Stage IV (23)	Head (7) Tail and/or Body (18)	N/A	US	HIFU	Pulsed wave HIFU, FEB- BY02 HIFU System	None
Gao, 2013 [60]	Prospective	39	58*	N/A	Head (7) Tail and/or Body (32)	N/A	US	HIFU	Continuous wave HIFU, Model-JC HIFU System	Chemotherapy and/or radiotherapy before HIFU (10) Chemotherapy concurrent with HIFU (25)
Anzidei, 2014 [24]	Prospective	7 (6 treated)	67	Stage III (7)	Body (7)	Tumor volume mean 20 +/- 5.6 mL	MRI	HIFU	ExAblate 2100; InSightec	Chemotherapy and/or radiotherapy before HIFU (7) Previous failed celiac plexus alcoholization (7) Continued chemotherapy after HIFU (7)
Sofuni, 2014 [61]	Prospective	30	64	Stage III (16) Stage IV (14)	Head (13) Unicinate (4) Body (9) Tail (1) Other (3)	Tumor size mean 31.7 +/- 1.7 mm	US	HIFU	Pulsed wave HIFU, FEB- BY01 HIFU System	Pre HIFU Operation (3) Chemotherapy (28) Radiation therapy (4) Interventional radiology (5) After HIFU Chemotherapy (24) Operation (2) Intervention radiology (5)
Marinova, 2016a [23]	Prospective	13	66.2	Stage III (5) Stage IV (8)	Body (12) Tail and/or Body (4) Head and Body (5)	Tumor volume range 12.6–61.8 mL, mean 34.3 +/- 17.9 mL	US	HIFU	Continuous wave HIFU, Model-JC HIFU System	Chemotherapy previous and concurrent with HIFU (10) Radiotherapy and surgery (1) Non therapeutic laparotomy (5) Plastic or metal stents for cholestasis (2) Percutaneous biliary drainage (1)
Li YJ, 2016 [34]	Prospective	16	62.3	N/A	Head (9) Body (7)	Mean max diameter 3.7 cm	US	HIFU+ Radiotherapy	N/A	N/A
Li X, 2016 [49]	Retrospective	120 (61 treated with HIFU)	50.13	N/A	Head (31) Other (30)	N/A	US	HIFU+ Chemotherapy	Continuous wave HIFU, Model-JC HIFU System	N/A
Strunk, 2016 [62]	Prospective	15	66.9	Stage III (6) Stage IV (9)	Head/Body (3) Body (5) Head (7)	N/A	US	HIFU	Continuous wave HIFU, Model-JC HIFU System	Pre HIFU Chemotherapy (13) Non therapeutic laparotomies (5) Surgery radiotherapy (1) Radiotherapy (2) Concurrent chemotherapy (13) Biliary drainage (4)
Lv, 2016 [48]	Prospective Randomized	45 (23 treated with HIFU)	59	Stage III (29) Stage IV (16)	Head (22) Tail and Body (23)	Tumor size range 8.1x7.5x5.8–2.6x2.5x1.8 cm	US	HIFU+ Chemotherapy	Continuous wave HIFU, JC200 HIFU System	N/A
Marinova, 2016b [36]	Prospective	20	68	Stage III (6) Stage IV (12)	N/A	Tumor volume 35.2 +/- 18.6 mL	US	HIFU	Continuous wave HIFU, Model-JC HIFU System	Concurrent chemotherapy (16) Chemotherapy after HIFU (2) Metal or plastic bile duct stent (6)

* median age

### Clinical outcome

**Fig. 2:**
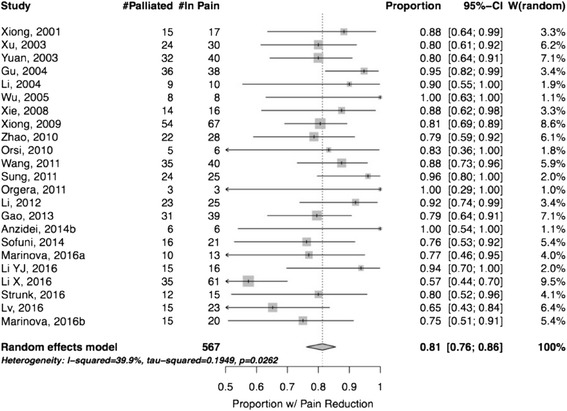
Random Effects Model – Studies included in the analysis. Proportion of patients with pain reduction

Among 639 patients treated with HIFU, 567 complained of pancreatic pain before the treatment. After HIFU, 459 patients experienced a partial or complete pain relief (Table 2). The random effects estimate of proportion of patients with pain reduction was 0.81 (95% CI: 0.76–86) (Fig. 2). Based on this result, we can conclude that 81% of patients may expect to have pain relief after HIFU treatment, notwithstanding that we have to take into consideration the variability among the studies. Therefore, the lower boundary of 76% is a more conservative, and probably more valid, estimate of the true value.

**Table 2:** Summary of the results of the included studies on HIFU therapy in pancreatic cancer

Author, Date	Pain Evaluation	Number of patients with pain at baseline	Number of patients with pain relief	% Patients with pain reduction	Pain Scale 0–10		HIFU related adverse effects (#pt)	
					Before HIFU	after HIFU	Minor	Major
Xiong, 2001 [53]	Pain Scale	17	15	0.88	7 +/- 2.1	3 +/- 1.5	None	Jaundice (1)
Xu, 2003 [54]	Pain Scale	30	24	0.80	5.6 +/- 3.2	2.0 +/- 1.9	Dilation of pancreatic duct with steatorrhea (3)	None
Yuan, 2003 [55]	Pain Category	40	32	0.80	N/A	N/A	None	None
Gu, 2004 [21]	N/A	38	36	0.95	N/A	N/A	N/A	N/A
Li, 2004 [22]	Pain Scale	10	9	0.90	N/A	N/A	Skin burn II (1)	None
Wu, 2005 [32]	Drug Needs	8	8	1.00	N/A	N/A	None	None
Xie, 2008 [56]	N/A	16	14	0.88	N/A	N/A	Skin burn (1)	Jaundice aggravation(1)
Xiong, 2009 [44]	Pain Scale	67	54	0.81	N/A	N/A	Skin burn II (3), Subcutaneous sclerosis (6), Pancreatic pseudocyst (1)	None
Zhao, 2010 [45]	VAS + Use of opioids	28	22	0.79	N/A	N/A	None	None
Orsi, 2010 [57]	N/A	6	5	0.83	N/A	N/A	None	Portal Vein Thrombosis (1)
Wang, 2011 [33]	Pain Scale	40	35	0.88	N/A	N/A	None	None
Sung, 2011 [35]	VAS	25	24	0.96	4.9 +/- 1.1 range 4–9	2.1 +/- 1.1 range 0–5	Mild abdominal pain (16), Transient pancreatitis (7), Transient fever (3), Severe abdominal pain with vomiting (2)	Pancreaticoduodenal fistula (2),Skin burn II (1), Skin burn III (1)
Orgera, 2011 [58]	Use of opioids	3	3	1.00	N/A	N/A	None	None
Li, 2012 [59]	Pain scale	25	23	0.92	4.6 +/- 2.1	2.2 +/- 0.9	Skin burn I (3)	None
Gao, 2013 [60]	Pain Scale	39	31	0.79	N/A	N/A	None	None
Anzidei, 2014 [24]	Pain Scale	6	6	1.00	7 +/- 1	3 +/- 1	None	None
Sofuni, 2014 [61]	Pain Scale	21	16	0.76	N/A	N/A	Mild pancreatitis (1) Pseudocyst formation (2)	None
Marinova, 2016a [23]	Pain Scale	13	10	0.77	N/A	N/A	Mild to severe abdominal pain (7), Skin burn II (1), Induration subcutaneous fat tissue (1), Local edema (6), Increase in pancreatic lipase (3)	Severe abdominal pain requiring hospitalisation (1)
Li YJ, 2016 [34]	Pain Scale	16	15	0.94	5.1 +/- 2.2	3.3	None	None
Li X, 2016 [49]	Pain Scale	61	35	0.57	6	N/A	Slight skin burns	None
Strunk, 2016 [62]	Pain Scale + Use of opioids	15	12	0.80	N/A	N/A	Transient subcutaneous edema (9), Skin burn II (1), Subcutaneous sclerosis (1), Lipase increase (3)	None
Lv, 2016 [48]	Memorial Pain Assessment Card	23	15	0.65	N/A	N/A	None	None
Marinova, 2016b [36]	Pain Scale	20	15	0.75	3.75 +/- 2.07	1.60 +/- 1.35	Mild severe abdominal pain (13), Cutaneous/subcutaneous edema (11), Subcutaneous tissue induration (1), Skin burn IIa (1), Increase in lipase (3)	None

**Fig. 3:**
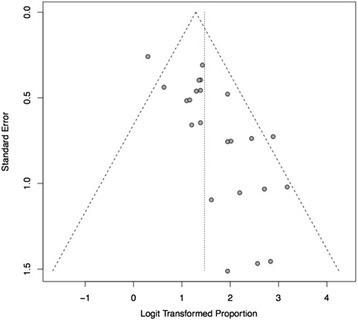
Funnel plot demonstrating possible but not statistically significant publication bias in assessment of pain (*P* > 0.05). -Dashed diagonal lines indicate 95% CI

The I^2^ of the included studies was 40% (95% CI: 1–64%). This result indicates that multiple effect sizes are possibly present, most probably because of the considerable variability in patients, treatments and other parameters in the publications. In fact, the Q test p-value was 0.026, confirming a significant heterogeneity among studies, as shown in Fig. 2. The funnel plot (Fig. 3) suggests a possible publication bias, with small less-successful studies missing, but the test of asymmetry was not statistically significant (*p* = 0.054).

Pain evaluation was heterogeneous among the studies, and the follow up periods were not consistent, with different or not specified assessment intervals. Eighteen studies used a quantitative estimate of the pain, adopting a numerical scale, VAS (Visual Analog Scale) or NRS (Numerical Range Scale) ranging from 0 to 10, with ten defined as the maximum pain experienced and 0 no pain reported. The associated use of painkillers was variably included in the definition of pain relief; although most of the patients reduced or discontinued the use of analgesic medications, it is not possible to quantify this rate due to heterogeneous reporting of the details of opioid analgesia in these papers.

The tumor response was not part of the statistical analysis since there were no uniform criteria, methodology and timing of evaluation among the studies. The modality of assessment and the number of patients with a tumor response after ultrasound ablation are summarized in Table 3. The most common criteria for the evaluation of a positive response were: changes in grey scale on US, RECIST (Response Evaluation Criteria in Solid Tumors) guidelines, WHO criteria, lack of contrast enhancement, lack of vascularity and reduction in size. With MRgFUS the Non Perfused Volume on MRI images was evaluated using 60% as threshold to define an efficacious treatment. Excluding two studies that did not report the data, 74% of the patients treated with HIFU had a positive tumor response.

The most commonly encountered mild adverse events following HIFU were mild to severe abdominal pain (*n* = 38, 5.9%), followed by edema (*n* = 26, 4.1%) and first and second degree skin burns (*n* = 11, 1.7%). Only eight cases of severe complications were reported (1,2%). (Table 2).

**Table 3:** Tumor response

Author, date	Tumor response			Complete response	Partial response	Stable disease	Progressive disease
	Imaging evaluation method	Parameter evaluated	Result #pt				
Xiong, 2001 [53]	US	hyperechogenicity	21				
Xu, 2003 [54]	US	hypovascularity	12				
Yuan, 2003 [55]	CT, US, CDFI	N/A	36	8	28	0	4
Gu, 2004 [21]	CDFI	N/A	N/A				
Li, 2004 [22]	CT, US,CDFI, pathological analysis only in the effective	reduction in tumor size, hyperechogenicity, blood flow decrease/disappearance	9				
Wu, 2005 [32]	CT or MRI	tumor reduction rate (range 20–70%)	8				
Xie, 2008 [56]	US	hyperchogenicity	15				
Xiong, 2009 [44]	CT or MRI	absence of perfusion	64				
Zhao, 2010 [45]	CT	RECIST	32	2	15	15	5
Orsi, 2010 [57]	PET/CT, CT or MRI	focal uptake of FDG, low attenuation at the ablation site without contrast enhancement at the edges	5				
Wang, 2011 [33]	CT	decreased enhancement	35	0	7	28	5
Sung, 2011 [35]	MRI	stack model (unenhanced area)	46				
Orgera, 2011 [58]	PET/CT or MRI, US, CT	lack of contrast and enhancement of metabolic activity	3				
Li, 2012 [59]	US, CT	hyperechogenicity, and hypovascularity(US), tumor necrosis and reduction (CT)	18				
Gao, 2013 [60]	CT or MRI	decrease or disappearance of blood supply in target region and circular enhancement in tumor periphery	30	0	5	25	9
Anzidei, 2014 [24]	CT and MRI	changes in density and intensity, contrast enhancement, non perfused volume (at least 60%)	6				
Sofuni, 2014 [61]	CT	WHO criteria	26	0	4	22	4
Marinova, 2016a [23]	US	lack of contrast enhancement	13				
Li YJ, 2016 [34]	MRI, CT, US	RECIST	11	0	7	4	5
Li X, 2016 [49]	CT	RECIST	16	1	15	N/A	N/A
Strunk, 2016 [62]	US, CT, MRI	tumor ablation rate (NPV/total volume)	8				
Lv, 2016 [48]	CT	RECIST	18	0	10	8	0
Marinova, 2016b [36]	CT and MRI	tumor volume reduction	N/A				

*FDG* flurodeoxyglucose, *NPV* non perfused volume, *CDFI* color doppler flow imaging, *RECIST* response evaluation criteria in solid tumors

### HIFU Technique

Preoperatively, a medical history, physical examination and biochemical laboratory blood tests were collected. The preparation of the patient differed among the studies. The most common procedures are reported. Before HIFU the patient underwent bowel preparation with 12–24 h fasting. Abdominal skin was prepared in order to avoid local skin burns with shaving and cleaning of the area. A pad located between the transducer and the patient’s abdomen was used to displace bowel loops from the US beam pathway. Additional procedures were: laxatives, liquid diet, traditional Chinese medication [21, 22], and a stomach tube to administer antifoaming agents and bind air bubbles [23]. If necessary, biliary stenting or a cholecystojejunostomy was performed to prevent or relieve the presence of obstructive jaundice.

Most of the studies included used US-guided HIFU devices, either the JC Model (Chongqing HIFU Technology Co, Ltd., Chongqing, China) or the FEB-BY HIFU system (Yuande Biomedical Engineering Limited Corporation, Beijing, China) (Table 1). Both systems use ultrasonography to visualize the tumor and to monitor tumor ablation; the main difference between these two is in the pattern of delivery and intensity of the ultrasound waves. The JC Model system delivers continuous wave focused ultrasound with high intensity, in the 5–20 kW/cm^2^ range, that allows a unique session treatment but requires sedation or general anesthesia and hospitalization of the patient. The FEB-BY system employs pulsed-wave focused ultrasound with low intensities, less than 3 kW/cm^2^. This results in the need for more than one treatment session per patient (from 4 to 7), but most treatments do not require sedation or hospitalization (Table 4) [18]. One study used an MRgFUS device (ExAblate 2100; InSightec, Haifa, Israel), performing all the procedures on a 3-T MRI scanner. The frequencies of the system range from 0.95 to 1.35 MHz, and the energy from 100 to 7200 J. The treatment was performed under general anesthesia with controlled respiration to overcome motion artifacts [24]. Overall, procedures were performed under different conditions of analgesia: 8 studies administered general anesthesia, 3 used sedative analgesia, 1 regional anesthesia and 3 patients had epidural anesthesia. 6 studies did not use any anesthetic and 5 papers did not report this information. MRgFUS was performed in a single treatment session, whereas the USgFUS was often delivered in several sessions, with the number of sessions varying based on the device used, general health of the patient and size of the tumor to be ablated.

Post-operatively, the skin was evaluated for development of skin burns, and biochemical blood tests were used to monitor for the development of pancreatitis. Depending on the study’s design and the authors’ preferences, several imaging modalities were used immediately after HIFU and in the post-treatment period to assess ablation and tumor response.

**Table 4:** HIFU Technical parameters

Author, date	HIFU device	HIFU Transducer features	Intensity and frequency	Acoustic output power	Continuous or pulsed wave	Number of sessions
Xiong, 2001 [53]	FEB-BY01 HIFU System	N/A	N/A	input power: 1–2 kW	pulsed	9.5 average, max 15
Xu, 2003 [54]	FEB-BY01 HIFU System	N/A	N/A	input power: 1–2 kW	pulsed	6.5 average, max 12
Yuan, 2003 [55]	FEB-BY01 HIFU System	effective treatment depth 3.5–14.0 cm; practice focused sphere 0.3 × 0.3 × 0.8 cm; effective focused sphere of 0.6 × 0.6 × 0.6 cm	N/A	1–2 kW	pulsed	40 patients received in total more than 280 HIFU treatments (2–4 times for smaller tumour focus)
Gu, 2004 [21]	FEB-BY01 HIFU System	depth of effective therapy 2–15 cm; actual focus measurement 0.3 × 0.3 × 0.8 cm; effective focus of 0.6 × 0.6 × 1 cm	N/A	1–2 kW average: 1.5 kW	pulsed	6 average (range 3–14)
Li, 2004 [22]	FEB-BY02 HIFU System	effective therapy depth of 2–15 cm; practice focused sphere of 0.3 × 0.3 × 0.8 cm; effective focused sphere 0.6 × 0.6 × 1 cm	N/A	1–2 kW	pulsed	8.4 average (range 5–12). Patients with abdominal and back pain got abdominal ganglion treatment 1–2 times per patient
Wu, 2005 [32]	Model-JC HIFU System	12 cm diameter; focal length 13.5 cm; focal region: 9.8 mm along beam axis, 1.3 mm in transverse direction	0.8 MHz; Acoustic focal peak intensity: 10 to 15 kW/cm2	N/A	continuous	1.5 average (2 patients had 2 sessions, 6 patients had 1 session)
Xie, 2008 [56]	HIFUNIT-9000 HIFU System	effective therapy depth: 17 cm; focused sphere: 0.3 × 0.3 × 0.8 cm	1 MHz	maximum output power: 600 W in the study: 200–300 W	continuous	4.25 average (range 2–8)
Xiong, 2009 [44]	FEB-BY HIFU System	overall aperture 37 cm; focal length 26 cm; -6 dB focal dimensions: 0.8 cm in length, 0.3 cm in diameter	1.04 MHz	250–430 W	pulsed	4–10 sessions
Zhao, 2010 [45]	HIFUNIT-9000 HIFU System	effective therapy depth 2–15 cm; practice focused sphere 0.3 × 0.3 × 1 cm	N/A	Input power: 3 kW/cm2	continuous	Gemcitabine on days 1, 8 and 15, and multiple HIFU sessions on days 1, 3 and 5. The combined treatment repeated every 28 days
Orsi, 2010 [57]	Model-JC HIFU System	20 cm diameter; focal length 15 cm	0.8 MHz	200–400 W	continuous	single session
Wang, 2011 [33]	Model-JC HIFU System	20 cm diameter, focal length 13.5 cm; focal region: 8 mm along beam axis, 1.5 mm in transverse direction	0.85 MHz	mean power range: 117–388 W median: 247 W	continuous	single session
Sung, 2011 [35]	Model-JC HIFU System	20 cm diameter; system operated by using one of several therapeutic transducers with focusing lengths that varied from 9 to 16 cm (13.7 cm focusing length most used in the study)	0.8 MHz (either 0.8 or 1.6 MHz for each focal length, but 0.8 most commonly used)	140–240 W (200 W most commonly used)	continuous	single session
Orgera, 2011 [58]	Model-JC HIFU System	diameter 20 cm; focal length 15 cm	0.8 MHz	60–400 W	continuous	single session
Li, 2012 [59]	FEB-BY02 HIFU System	aperture of 37 cm; focal distance 25.5 cm; focus has a 6 dB beam width of 1.6 mm and an axial length of 1 cm; effective therapy depth 2-15 cm	1 MHz	400–1000 W mean: 586 +/- 78.4 W	pulsed	1.2 average (19 patients had 1 session, 6 patients had 2 sessions)
Gao, 2013 [60]	Model-JC HIFU System	diameter 20 cm; focal length 13.5 cm	0.85 MHz	N/A	continuous	33 patients had 1 session, 4 patients had 2 sessions, and others more than 2 sessions
Anzidei, 2014 [24]	ExAblate 2100; InSightec	diameter 12 cm; radius of curvature 16 cm; focal distance 6–20 cm	0.95–1.35 MHz	N/A		single session
Sofuni, 2014 [61]	FEB-BY02 HIFU System	aperture of the ultrasound array 37 cm; radius of curvature 25.5 cm	1.1 MHz	input electric power: 0.5–2 kW	pulsed	2.7+/-0.1 SD
Marinova, 2016a [23]	Model-JC HIFU System	20 cm diameter; focal length 15 cm	0.8 MHz	range: 80–400 W average: 344 +/-72 W (200–400)	continuous	single session
Li YJ, 2016 [34]	N/A	N/A	0.8 MHz	300 W	N/A	N/A
Li X, 2016 [49]	Model-JC HIFU System	N/A	N/A	N/A	N/A	single session
Strunk, 2016 [62]	Model-JC HIFU System	diameter 20 cm; focal length 15 cm	0.8 MHz	200–400 W	continuous	single session
Lv, 2016 [48]	JC200 HIFU System	focus 14.7 cm	0.97 MHz	average: 350 W	continuous	single treatment expected, but additional treatments can be added when necessary
Marinova, 2016b [36]	Model-JC HIFU System	diameter 20 cm; focal length 15 cm	0.8 MHz	N/A	continuous	single session

## Discussion

The origin of pain from pancreatic cancer is multifactorial, resulting from tumor infiltration of nerves, tumor mass compression and inflammatory reaction elicited by the malignancy [25, 26]. The mechanisms by which HIFU relieves pain are not completely understood. Three possibilities have been proposed: 1) thermal damage to the nerves innervating the tumor, 2) fibrosis and shrinkage of the tumor after ablation, resulting in reduced mass effect, and 3) the inactivation of the fibers of the celiac plexus that normally transmit the pain sensation centrally [10].

Our study suggests that HIFU is a very effective mean of relieving pain in patients with pancreatic cancer. Despite the heterogeneity in the studies published in the literature, 81% of patients had a partial or total decrease of pain following the treatment. Case reports published in the literature, excluded from our analysis because of their small sample size, are consistent with our findings on the efficacy and safety of HIFU for pain palliation [27–31].

Not all papers reported the duration of pain relief. The longest follow up period was reported by Wu et al., with no pain progression seen in a up to 17 months, and Anzidei et al., with pain alleviation persisting at 6 months [24, 32]. Wang et al. and Li YJ et al. reported a median pain relief time of 10 weeks and 5.6 months, respectively [33, 34]. Other studies with short-term follow up (≤3 months) confirm the relief of pain [23, 35, 36].

The main treatment proposed when opioids fail in pain control is currently neurolytic celiac plexus blockade (NCPB). NCPB involves percutaneous or endoscopic injection of anesthetics and neurolytic substances (Ethanol or Phenol) along the celiac plexus in order to interrupt nociceptive transmission [25]. The actual efficiency in the reduction of pain is variable and, in some studies, it has been questioned [13, 37]. Although response rates as high as 70–90% were initially reported [12], a subsequent meta-analysis of five randomized control trial demonstrated only a 6% reduction in pain scores after neurolytic celiac plexus blockade [13]. NCPB has also been reported to result in reduced opioid use and related side effects [37–39]. However, Wong et al., in a double blinded randomized control trial that compare NCPB to a placebo, found no difference in opiate use or side effects between the two groups [40]. Most of the studies found a short duration of analgesia, lasting about 3 months [38, 39, 41, 42], with repeated NCPB demonstrating reduced efficacy (29% in repeated vs 67% after initial block) [42]. Indeed, the most commonly encountered side effects in NCPB include: local pain (96%), transient diarrhea (44%) and hypotension (36%) [12, 41]. More severe adverse events occur in 2% of patients, including but not limited to: pneumothorax, shoulder, chest and pleuritic pain. One percent of these complications are neurological, with occurrence of paraplegia representing the major concern [12, 41].

Focused ultrasound technology appears to be an attractive alternative because it is non-invasive, provides rapid pain relief and has a high safety profile [10], it has also been used successfully after NCPB has failed [24]. It offers the potential for a multimodal therapeutic approach for patients with pancreatic cancer, providing pain palliation and the possibility of local tumor control and increased local delivery of chemotherapeutic agents [15]. Compared with neurolytic celiac plexus blockade, the rate of adverse effects in our studies was considerably lower. HIFU has a high safety profile, with only eight cases of severe complications (1.3%) reported in our analysis. In a study published by Jung et al., the adverse events after HIFU treatment for hepatic and pancreatic cancer were listed. Among them, there were skin redness, edema and pain in the treated region; for the 35 patients with pancreatic cancer treated with HIFU, 3rd-degree skin burn (*n* = 1, 2.9%) and fistula formation between the tumor and the duodenum were listed as major complications (*n* = 3, 8.5%) [43]. In our studies, the most common side effect encountered is mild to severe abdominal pain, followed by skin burns of various degree. The abdominal pain is usually self-limited, with only one case requiring hospitalization. The most feared adverse event following HIFU is bowel perforation due to interposition of the intestinal loops along the ultrasound beam pathway; two cases of pancreaticoduodenal fistula (0.3%) occurred among our patients. Eight patients developed mild or transient pancreatitis (1.3%) classified as minor complication, in nine further patients there was a mild increase of lipase on blood analysis without any clinical signs of pancreatitis (1.4%). Unlike chemotherapy, HIFU does not have systemic side effects that limit dose or number of applications and in contrast to radiation therapy, it is not a risk for poor wound healing or secondary malignancies.

In these studies, local tumor control achieved after HIFU was assessed through different imaging methods. Among 639 patients who underwent HIFU for pancreatic cancer, 455 had a positive tumor response excluding two studies not reporting this data (Table 3). These studies reveal that tumor response is not always correlated to pain relief, suggesting that complete ablation is not necessary for pain relief. Xiong et al. observed pain improvement in 88% of patients who had an objective tumor response but also in 76.2% of patients who did not [44]. Similarly, Zhao et al. observed pain relief in 88.2% of patients with a tumor response and in 35% of patients with stable or progressive disease [45]. Tumor size reduction does not appear to be a sensitive way to evaluate HIFU efficacy, neither in terms of the effect on pain relief nor for the evaluation of successful ablation. Indeed, in the short term, despite the reduction in pain, the volume of the mass may appear unchanged or increased due to local edema [46, 47].

Pancreatic adenocarcinoma is relatively hypovascular and is surrounded by a thick fibrous ring that limits the penetration and diffusion of chemotherapeutic agents, accounting in part for the poor responsiveness to pharmacological treatment. Recent studies have demonstrated that HIFU may have a synergistic effect with chemotherapy, boosting the drug concentration in the tumor and reducing the systemic toxicity. The underlying mechanisms proposed are increased permeability of vascular endothelial cells and enhanced diffusion of the chemotherapeutic agent under the radiation force from the ultrasound field [15]. Among the studies included in our analysis, two compare the combination treatment of HIFU plus chemotherapy to chemotherapy alone. Lv et al. conclude that the difference in pain relief between the two groups was significantly improved in the HIFU combination group (65.2% vs 31.8%); although the disease control rate was also higher in the HIFU group (78.2% vs 59.0%), this was not statistically different. Moreover, the combination therapy was associated with considerably improved survival rates at 6 months (73.9% vs 40.9%, *P* < 0.05), but not at 12 months (13.0% vs 4.5%, *P* > 0.05) [48]. Li X et al. reported similar results with a significant better overall survival (10.3 months vs 6.6 months), PFS (Progression Free Survival: 5.1 months vs 2.3 months), objective tumor response (26.2% vs 8.5%), and remission rate of pain (57% vs 20%) [49].

Good control of pain relief has a significant impact on the quality of life of the patient, but further studies are needed to assess the potential that this may have on survival. Survival benefit following HIFU has been previously reported in the literature. Vidal et al. observed an unexpectedly prolonged survival time for patient with stage III and stage IV pancreatic carcinoma treated with HIFU and chemotherapy, with the longest survival of 3.4 years. Moreover, the estimate of the survival was surprisingly high, with 33.5% pancreatic cancer patients still alive at 4.2 years [50]. HIFU was combined with gemcitabine in this study; considering the absence of a control arm, it is possible that the improved survival partially reflects patients that responded well to chemotherapy. Most of the studies included in our analysis have not been designed to assess the survival benefit of HIFU in the treatment of advanced pancreatic cancer. The median overall survival is reported in 12 studies; the range is 7–25 months with a median value of 10 months. These data are very heterogeneous with no definition of a starting point nor duration of follow-up. Moreover, some of the papers report survival after HIFU-only while others after HIFU combined with other treatments. Clearly, further research is needed to validate these results.

The main limitation in our study is the lack of randomized controlled trials and the considerable heterogeneity in the data reported by the single papers, which sometimes made comparison of the results not feasible. The most important differences were in the evaluation of pain, assessment of tumor response and technique used for the treatment. It was not possible for us to estimate quantitatively the reduction of pain following HIFU, as most of the papers did not use a numerical scale to assess the difference at baseline and follow up. Moreover, the timing of pain evaluation was not consistent, while precise intervals would allow an estimate of pain relief in the long term. A considerable heterogeneity was observed in the use of analgesic drugs, in terms of need of pain medications after treatment, opioid or non-opioid use, and eventual doses required. Some studies defined pain relief considering only the absolute decrease in pain, while others included the change in analgesic drugs need. Likewise, the tumor response evaluation differed in the criteria, timing and methodology used to evaluate it. A more consistent follow up time is necessary to assess longer term results and to address the potential survival benefit following HIFU. Uniformity is needed in the inclusion criteria used and in the description of the characteristics of the tumor treated (histological type, location, size, stage) in order to make data more homogeneous and comparable.

Most of the current literature reports experience with USgFUS, and few reports suggest the feasibility and safety of MRgFUS. The US guided methodology uses ultrasound for both the detection and ablation of the lesion, allowing identification of potential obstructions in the US beam pathway, such as air. USgFUS has some limitations: its contrast resolution may not be adequate to depict accurately the borders of the lesion, it lacks a real-time temperature monitoring to ensure adequate ablation, and it is operator dependent. MRgFUS is promising, because of improved tissue contrast allowing definition of the tumor and surrounding structures and because of real time MR thermometry, which allows better targeting and monitoring of the ablated region [16, 46, 51, 52].

Even though most of the studies were performed with US guided HIFU, standardization of energy, power and technical parameters are lacking, and still needed to obtain the best results at the minimum risk for the patient. The studies included used two different US systems; indeed, the biological effect of continuous wave HIFU can have considerable differences from the pulsed wave focused ultrasound in terms of biological effects on the tissue, interaction with other therapeutic regimens and clinical response of the patient. Not all papers specified if HIFU was applied alone or with concurrent chemotherapy/radiotherapy. In the future, these data need to be specified to better discriminate the potential of HIFU as single therapy and the effects of combination therapies on tumor response.

Therefore, there is need for uniformly designed studies in order to determine the data necessary to report in each trial, in order to objectively evaluate the treatment results. A clinical registry of the results of HIFU treatment of pancreatic cancer is planned. This will provide an analytical tool useful to assess the eventual benefit of HIFU on the overall survival and pain palliation in pancreatic cancer, which is still a poorly treated aggressive tumor bearing a poor prognosis.

## Conclusions

Although the literature is heterogeneous, our study supports that High Intensity Focused Ultrasound is a potent tool for pain palliation in unresectable pancreatic cancer. The potential role of HIFU requires further well designed studies to confirm its efficacy, safety and advantages compared to other palliative techniques.

## Abbreviations

HIFU: High intensity focused ultrasound
MRgFUS: MR guided focused ultrasound
NCPB: Neurolytic celiac plexus blockade
USgFUS: US guided focused ultrasound
